# Advances in technical methods and applications of subretinal injections in experimental animals

**DOI:** 10.3389/fvets.2025.1574519

**Published:** 2025-04-30

**Authors:** Chunyan Song, Yuke Ji, Yun Wang, Weihua Yang

**Affiliations:** Shenzhen Eye Hospital, Shenzhen Eye Medical Center, Southern Medical University, Shenzhen, China

**Keywords:** subretinal injection, animal model, ophthalmology, method, application, eyeball

## Abstract

The subretinal injection technique is an important intraocular drug delivery modality that allows access to the subretinal space to directly act on target cells or the administration of medications, markedly improving the therapeutic efficacy of ocular diseases. Subretinal injection in experimental animals is a commonly used manipulation method for investigating vitreoretinal diseases, particularly when gene therapy and cell therapy studies are involved. In this study, we conducted a systematic review on the injection methods, operation sites, post-injection indicators, as well as the progress and significance of subretinal injection in experimental animals, discussed and compared the advantages and disadvantages of the subretinal injection technique, summarized its specific application of subretinal injection in experimental animals, and explored the development and application of this new technology of subretinal injection, hoping to offer insights that may facilitate the further development of this technology.

## Introduction

The eye’s intricate structure comprises the eyeball wall, ocular contents, and appendages, with the eyeball wall divided into three layers: sclera, choroid, and retina. This complexity necessitates diverse methods of drug administration to treat various ocular conditions. Traditional methods such as topical application, intraocular injection, and systemic administration have their limitations, including poor penetration, potential for systemic side effects, and challenges in targeting specific ocular tissues. Therefore, the development of more precise and effective injection techniques has become crucial for advancing ocular therapy. In experimental animals, intraocular drug administration primarily involves intravitreal and subretinal injections, with subretinal injection being a particularly prevalent technique in retinal research. Compared with other delivery methods like intravitreal injection, subretinal injection techniques have its unique advantages. Firstly, it enables the direct delivery of therapeutic agents to the subretinal space, where the target cells reside, thereby maximizing the local drug concentration and minimizing systemic exposure. This is particularly advantageous for gene therapy and cell therapy, where precise targeting is crucial for therapeutic success. Secondly, subretinal injection allows for the controlled release of therapeutic agents, enabling sustained drug levels in the target tissue and prolonged therapeutic effects. This method involves the direct injection of drugs, cells, nucleic acids, small molecules, macromolecules, viruses, or biomaterials such as nanobeads into the subretinal space of experimental animals (1, 2). It is commonly applied in the treatment of retinal diseases such as macular degeneration (3, 4), funduscopic diseases (5, 6), retinal detachment (7), and certain infectious eye conditions (8). Subretinal injection plays a pivotal role in ophthalmology and neuroscience, significantly contributing to the study of ocular diseases, drug delivery, and gene therapy (9).

Advancements in subretinal injection technology for experimental animals have significantly enhanced the precision of drug delivery and therapeutic outcomes. Consequently, its clinical application has expanded. For instance, subretinal injection of anti-VEGF drugs is now a standard treatment for wet AMD (10), and ocular nanocarriers are in clinical trials (11). Furthermore, subretinal injection is employed for delivering viral vectors across various animal models (12, 13). This technology also holds promise for gene therapy and regulation, facilitating the delivery and expression of gene therapies through the injection of vectors carrying specific genetic information into the subretina (9). Technological innovations, such as precision injection robots (14) and intraoperative OCT imaging (15, 16), have further improved safety and precision, reducing surgical risks and improving treatment efficacy.

The subretinal injection technique is vital for ophthalmic research and clinical practice. Its ongoing evolution is anticipated to open up new possibilities for the treatment and study of ocular diseases. Nevertheless, to ensure its reliability and effectiveness in medical applications, there is a critical need to improve the standardization, safety assessment, and clinical validation of this technique in experimental animals. This review delineates the operational methods, procedures, advantages, and drawbacks of subretinal injection in experimental animals. It also encapsulates its application in animal modeling and therapy and explores the development and application of novel subretinal injection techniques.

## Methods of subretinal injection in experimental animals

**Figure 1:**
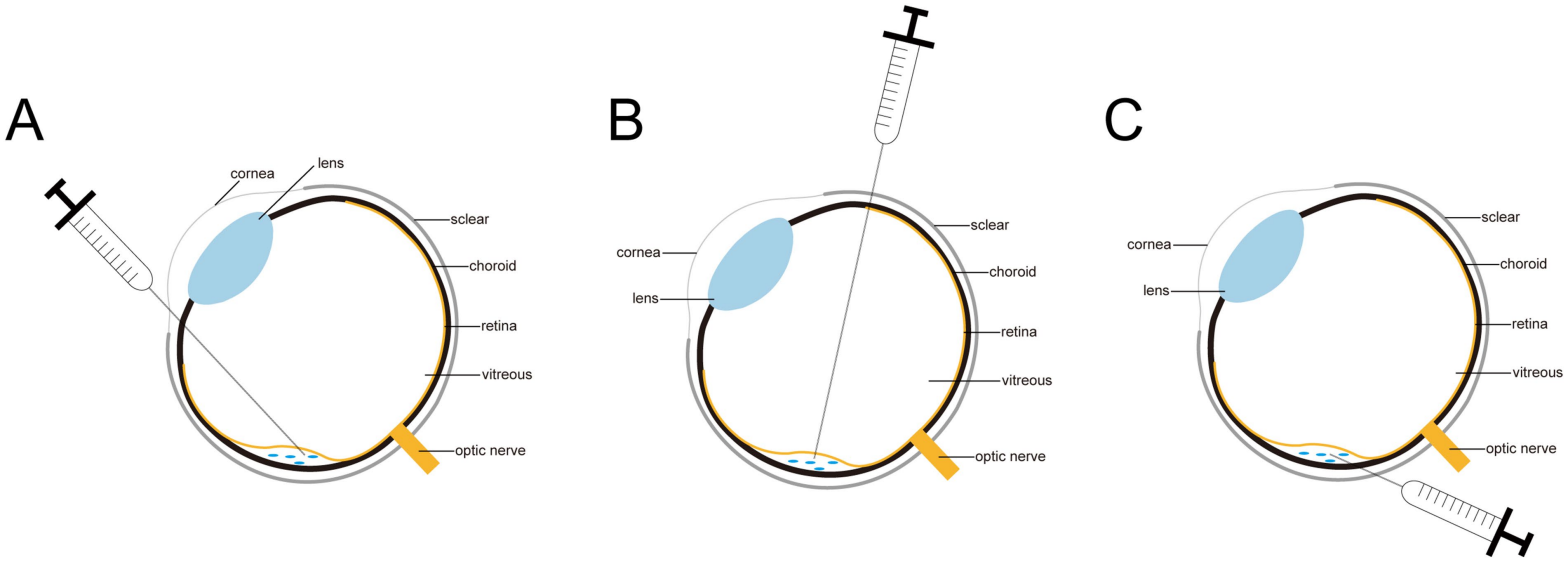
Diagram of three subretinal injections. **(A)** The corneal approach. **(B)** The transscleral posterior approach. **(C)** The pars plana approach.

**Figure 2:**
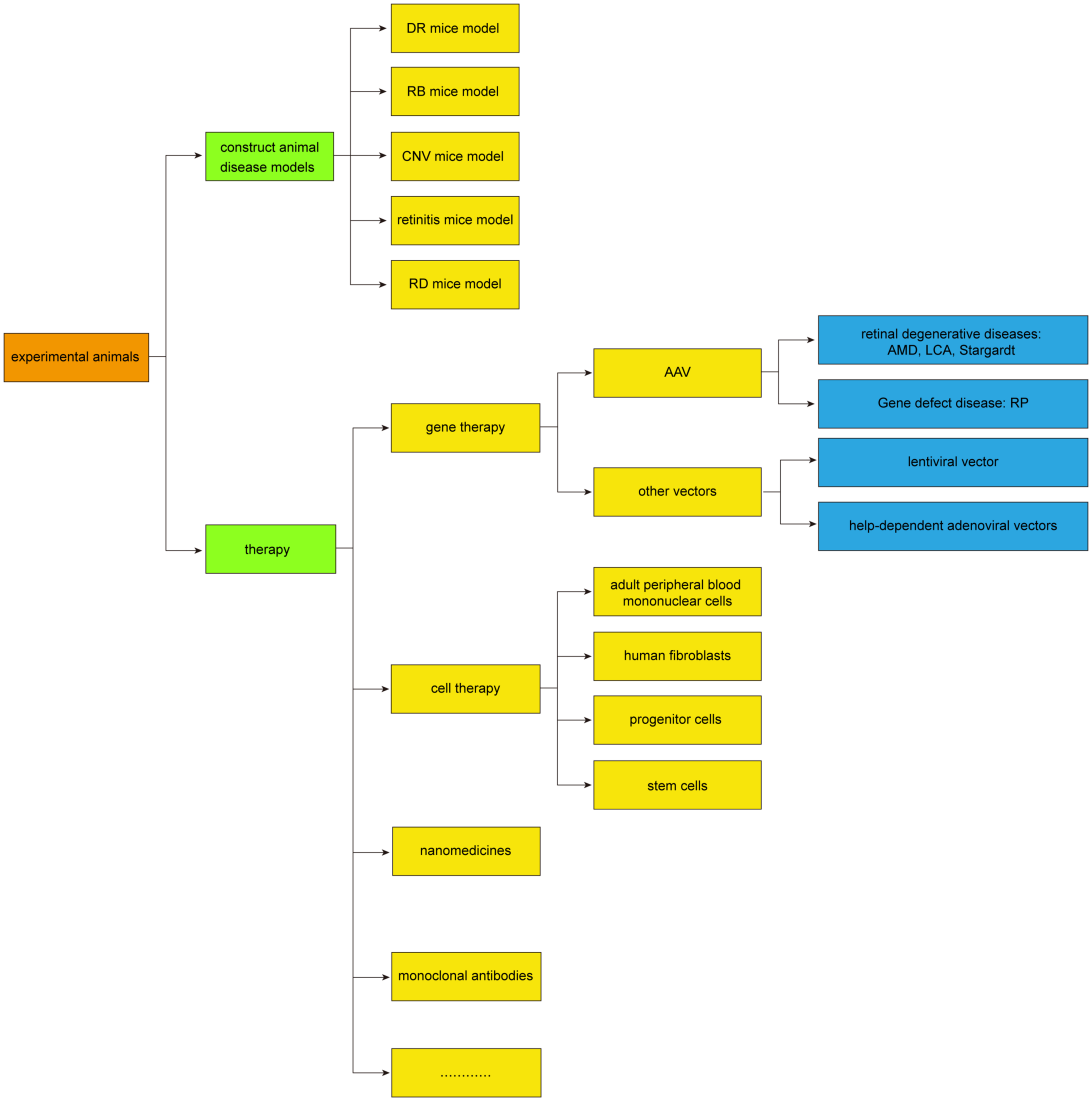
The general flow of subretinal injection.

In experimental animals, subretinal injection is performed using three primary techniques: transcorneal, posterior scleral, and pars plana subretinal injection, as depicted in Figure 1. The detailed procedures for each method are outlined below. Additionally, Figure 2 illustrates the overall process of subretinal injection.

### Transcorneal subretinal injection

The transcorneal subretinal injection method is a widely adopted technique for delivering substances to the subretinal space of rodent eyes. The procedure begins with general anesthesia, administered via intraperitoneal injection of a mixture containing 12.5 mg/kg mephenothiazine and 62.5 mg/kg ketamine. Following this, the pupil is dilated with 2.5% phenylephrine, and topical anesthesia is applied using 0.5% proparacaine. The eyelashes are trimmed to enhance visibility of the eye and fundus. A dissecting microscope is then utilized to monitor the pupillary dilation process. Once the anesthesia takes effect, the pupil is further dilated, and additional local anesthetic drops are applied. With the pupil fully dilated, the rat is positioned laterally under the microscope. A drop of 2.5% methylcellulose is placed on the cornea to facilitate visualization of the fundus. A 28-gauge hypodermic needle is carefully inserted nasally into the cornea, approximately 0.5 to 1 mm from the dilated pupil’s edge. The needle is then fully penetrated through the cornea into the anterior chamber, parallel to the anterior lens surface, ensuring at least 50% of its beveled surface is within the cornea to create an entry point for a 33-gauge blunt needle. The blunt needle’s tip is delicately advanced through the corneal puncture into the anterior chamber, taking care to avoid the iris and lens. The needle is then angled slightly nasally towards the posterior chamber, positioning the iris laterally and the lens medially. The lens is gently moved medially to allow the needle to reach the injection site. Resistance felt during needle advancement indicates successful penetration of the retina into the subretinal stroma. At this juncture, the syringe is held steady, and the contents are slowly injected over approximately 30 s by an assistant, leading to a visible retinal detachment. Post-injection, the needle is carefully withdrawn to minimize reflux of the injected material through the corneal wound. For successful subretinal injections, deep anesthesia should be maintained for 20 to 30 min. Real-time microscopic monitoring is essential, and full pupil dilation is mandatory to prevent complications. This method is unsuitable for neonatal mice due to their immature ocular structures and insufficient pupil dilation. Nonetheless, it is effective and safe for adult mice when executed by experienced practitioners (17).

It is straightforward to learn and requires minimal equipment and preparation. Typically, between 10 to 30 eyes can be injected per hour. Importantly, the procedure is designed to avoid damaging the lens, retina, or retinal pigment epithelium (RPE).

### Posterior scleral subretinal injection

Parikh et al. (18) investigated the posterior scleral subretinal injection method in mouse eyes, which offers a safer alternative to transretinal approaches. Mice were anesthetized with an intraperitoneal injection of a saline mixture containing 100 mg/kg ketamine and 8 mg/kg xylazine. Following anesthesia, the pupils were dilated with 2.5% phenylephrine eye drops, and the whiskers were trimmed to enhance visibility during the procedure. A 5 μL syringe was prepared with an injection volume ranging from 0.3 to 1.0 μL. The mice were positioned beneath a dissecting microscope with their eyes facing upwards to ensure clear visualization. The temporal conjunctiva was gently held with fine-tipped forceps, and a 90-degree circumferential incision was made through the conjunctiva and ocular fascia using curved Vannas scissors. The eye was rotated nasally, and connective tissue was carefully excised with forceps to expose the injection site approximately 0.5 mm temporal to the optic nerve, taking care to avoid the retro-orbital sinus. A small scleral incision was made at this site using a 22.5-degree ophthalmic blade, creating an opening sufficient for a 33-gauge needle. The 33-gauge needle, beveled at a 5–10° angle, was inserted into the scleral incision with the bevel facing the retina. The plunger was slowly depressed within approximately 3 s, maintaining even pressure throughout the injection. It is important to note that slight resistance indicates the needle is in the subretinal space, whereas no resistance suggests the retina has been pierced, and significant resistance indicates the needle has not penetrated the sclera or RPE. The needle was left *in situ* for several seconds to avoid reflux before being carefully removed. Following the injection, the eye was flushed with sterile buffered saline to ensure it returned to its normal position.

To ensure the procedure’s success, the depth of anesthesia was monitored to eliminate the corneal touch reflex, and the mouse’s body temperature was maintained at 37°C with a warming pad. Methylcellulose eye drops were applied post-anesthesia to prevent eye dryness and minimize the risk of anesthesia-induced cataracts. All instruments were sterilized using iodophor and ethanol or hot beads before surgery, and syringes were cleaned thoroughly with a suitable solvent and deionized water between injections. For enhanced visualization, a 0.01% fluorescein solution in 0.9% saline could be injected, and its distribution recorded using OCT fundus imaging. It is crucial to trim only the obstructive portion of the whiskers to preserve the mice’s sensory input.

The posterior scleral subretinal injection method allows for the observation of normal structural and functional contours of the subretinal space 4 weeks post-surgery. This technique has a high success rate, low exclusion rate, and minimal complications. It is safer as it avoids retinal perforation and vitreous penetration, limiting collateral damage to local sclerotomy-related injury and transient plasma retinal detachment. This method is highly effective at delivering viral vectors, drugs, stem cells, or induced pluripotent stem (iPS) cells into the subretinal space of mice with high efficacy, minimal damage, and rapid recovery. The complications and limitations of posterior scleral subretinal injection include potential scleral perforation, intraocular hemorrhage, and retinal detachment, albeit at lower rates compared to other techniques. Careful manipulation of instruments and precise needle placement are paramount to mitigate these risks. Additionally, the use of a warming pad to maintain body temperature and meticulous sterilization protocols contribute to reducing postoperative complications. Despite these potential complications, the posterior scleral subretinal injection technique remains a favored approach due to its enhanced safety profile and efficacy in delivering therapeutic agents to the subretinal space of mice.

### Pars plana subretinal injection

In animal models, the pars plana subretinal injection is a prevalent technique for delivering substances to the subretinal space. In a study (19), rats were anesthetized with an intraperitoneal injection of a ketamine and thiazide mixture and received local anesthesia with Obrocaine. The pupils were dilated using a combination of phenylephrine and tropicamide, and the anesthesia’s duration was approximately 30–50 min, providing ample time for the subretinal injection. Once the pupils were dilated, the anesthetized rats were positioned laterally under a surgical microscope. A 6 mm diameter rubber ring was placed on the corneal surface, and a drop of carbomer eye drop was applied inside the ring to enhance visualization of the fundus. A 27-gauge needle was then used to puncture the sclera temporally approximately 1–2 mm from the corneal limbus. The needle was inserted at a 45-degree angle behind the lens, beveled upward, and passed through the sclera into the vitreous body. At least 50% of the bevel was pushed through the sclera to create an opening sufficient for a 33-gauge blunt needle. The blunt needle’s tip was carefully inserted through the scleral puncture into the vitreous body. Upon feeling slight resistance, indicating the needle had reached the subretinal space, the procedure required cautious advancement to prevent lens damage. Subsequently, 4 μL of fluid was slowly injected into the subretinal space over 20 s, and the needle was gently withdrawn once the injection was complete.

Pars plana subretinal injection is one of the most commonly used techniques for subretinal injection. This technique avoids the macular region and critical structures in the central retina, thereby minimizing retinal damage and offering better protection of visual function. Additionally, the pars plana, located between the anterior and posterior poles of the eye, is a relatively flat area of the ocular wall, devoid of complex vascular and neural networks, which allows for minimal interference with surrounding tissues. This significantly reduces the risk of intraoperative bleeding and other complications. Overall, pars plana subretinal injection offers the benefits of being minimally invasive, highly precise, with rapid recovery and fewer complications. Despite its advantages, pars plana subretinal injection is not devoid of complications. One potential issue is intraocular bleeding, which can occur due to inadvertent injury to scleral blood vessels during needle insertion. Careful manipulation of the needle and meticulous attention to anatomical landmarks can minimize this risk. Additionally, cataract formation is a concern, particularly if the needle comes into contact with the lens during the procedure. However, advancements in needle design and injection techniques have significantly reduced the incidence of this complication. Retinal detachment is another potential risk, but it is usually manageable with prompt surgical intervention. Other less common complications include endophthalmitis, vitreous hemorrhage, and increased intraocular pressure. Despite these potential complications, pars plana subretinal injection remains a valuable tool in the arsenal of ophthalmic surgeons for the delivery of therapeutic agents to the subretinal space. The key to minimizing complications lies in meticulous preoperative planning, precise intraoperative technique, and vigilant postoperative care.

In summary, the three injection methods, including transcorneal subretinal injection, posterior scleral subretinal injection, and pars plana subretinal injection, each present a unique set of attributes. Transcorneal subretinal injection offers the advantage of direct visualization and ease of access, particularly suitable for anterior segment diseases. However, it may be accompanied by complications such as corneal edema and intraocular pressure spikes. Posterior scleral subretinal injection, on the other hand, allows for a more posterior approach, minimizing the risk of anterior segment complications. Yet, it requires a higher degree of surgical skill and may be associated with scleral perforation. Pars plana subretinal injection, as previously emphasized, stands out due to its reduced risk of retinal detachment and vitreous hemorrhage, which is particularly favored in gene and cell therapy applications where precise delivery to the subretinal space is crucial. However, it may involve more complex preoperative planning and postoperative monitoring.

In terms of application ranges, transcorneal injection is often used for anterior segment interventions, while posterior scleral and pars plana injections are more suited for posterior segment diseases. The selection of the injection method should be based on the specific disease, location, and therapeutic needs, along with the surgeon’s experience and preference. Operational challenges vary across methods. Transcorneal injection may face difficulties in maintaining intraocular pressure stability, while posterior scleral injection demands precise scleral localization. Pars plana injection, despite its advantages, may encounter challenges such as vitreous hemorrhage and endophthalmitis, necessitating vigilant preoperative and postoperative care.

In conclusion, each injection method has its own merits and drawbacks, and the choice should be tailored to the individual patient’s condition and treatment goals, ensuring the safest and most effective outcome.

## Assessment methods and common changes after subretinal injection

Experimental animals undergo various assessment methods following subretinal injection, including optical coherence tomography (OCT), electroretinography (ERG), color fundus photography (CFP), fluorescein fundus angiography (FFA), and retinal histologic sections (20, 21). These methods are crucial for monitoring changes in retinal morphology and function post-injection. Typically, a short period after subretinal injection, most mice experience retinal detachment, which gradually resolves as the retinal structure returns to normal over time (22). OCT examinations conducted by José et al. (23) at 30 and 240 days post-injection demonstrated spontaneous reattachment of the retina to the choroid. In cynomolgus monkeys injected with a balanced solution under the retina, ERG measurements revealed initial functional suppression followed by almost complete recovery within 9 days (24). Notably, 7 to 9 months post-injection, ERG amplitude was significantly affected compared to uninjected mice (1). In rabbits, a marked reduction in total retinal thickness, particularly the photoreceptor layer, was observed (25). Maya et al. (4) documented the formation of subretinal blebs and subsequent retinal reattachment through CFP and noted mild inflammatory reactions in some eyes via retinal histologic sections.

These observations indicate that subretinal injections significantly impact both the structure and function of the eye in animals. The extent of these effects is influenced by factors such as the injected dose, the distribution of the fluid, and the time elapsed since injection. These insights are vital for comprehending the pathogenesis of retinal diseases and for the development of innovative therapeutic strategies.

## Advantages and disadvantages of subretinal injections

Subretinal injection is a widely used technique in ocular treatment and research, offering distinct benefits and challenges. It allows for the precise delivery of drugs or therapeutic substances directly to the subretinal layer, enhancing the specificity and effectiveness of treatments for various ocular diseases. Stranak et al. (26) showed that direct subretinal cannula injection, without the need for vitrectomy, can achieve surgical goals in a shorter time frame with quicker recovery, without postoperative complications such as endophthalmitis, retinal detachment, or increased intraocular pressure. The rapid absorption of drugs into the retinal tissue through subretinal injection accelerates their arrival at the treatment site, optimizing therapeutic outcomes and minimizing discomfort and side effects (13). This method also circumvents the digestive system, preventing enzymatic degradation and preserving drug stability and efficacy. In the realm of gene therapy, subretinal injection has proven to be a safe and effective method of localized administration to the subretinal space in animal models, with minimal systemic toxicity and excellent tolerance (12, 27–30). Unlike intravitreal or choroidal injections, subretinal injection enables more targeted delivery, lowering the required dose and systemic exposure—especially advantageous for potentially toxic drugs.

Despite its advantages, subretinal injection is a highly technical procedure that necessitates specialized training and experience to avoid complications and ensure safety. Although it carries a risk of medically induced retinal detachment, the technique is generally associated with minimal trauma and rapid recovery of retinal structure and function, presenting an overall favorable safety profile (31). As with any form of injection, there is a risk of infection, and subretinal injections are no exception either. They may also lead to potential complications such as retinal detachment, requiring careful consideration, including the need for general anesthesia (32). Other risks associated with the transcorneal anterior injection route include total retinal detachment, mild corneal and lens clouding, anterior adhesions, iris hemorrhage, and external photoreceptor segment damage (17). These factors highlight the need for precision and caution in the application of subretinal injection, balancing its precision and rapid absorption benefits against the operational complexity and infection risk (Table 1).

**Table 1:** The advantages and disadvantages of subretinal injections.

	Advantages	Disadvantages
1	**Precision drug delivery:** Ability to deliver drugs or therapeutic substances directly to the subretinal layer to achieve more accurate treatment effects.	**High technical requirements**: It requires professionally trained and experienced operators to perform, and improper operation may lead to complications and injuries.
2	**Rapid absorption**: The drug can be quickly absorbed by the retinal tissue and quickly reach the treatment site.	**Safety risks**: There is a risk of iatrogenic retinal detachment, although it is associated with minimal trauma and early recovery of retinal structure and function.
3	**Improve treatment effectiveness**: Maximize treatment effectiveness and minimize discomfort and side effects.	**Potential complications**: Retinal detachment and other potential complications may occur, and general anesthesia may be required.
4	**Gene delivery**: As a local delivery method, the vector is relatively safe, has non-systemic toxicity, and is well tolerated.	**Risk of eye injury**: Total retinal detachment, mild corneal and lens opacities, anterior synechiae, iris hemorrhage, and outer photoreceptor segment damage.

## Application of subretinal injection in experimental animals

**Figure 3:**
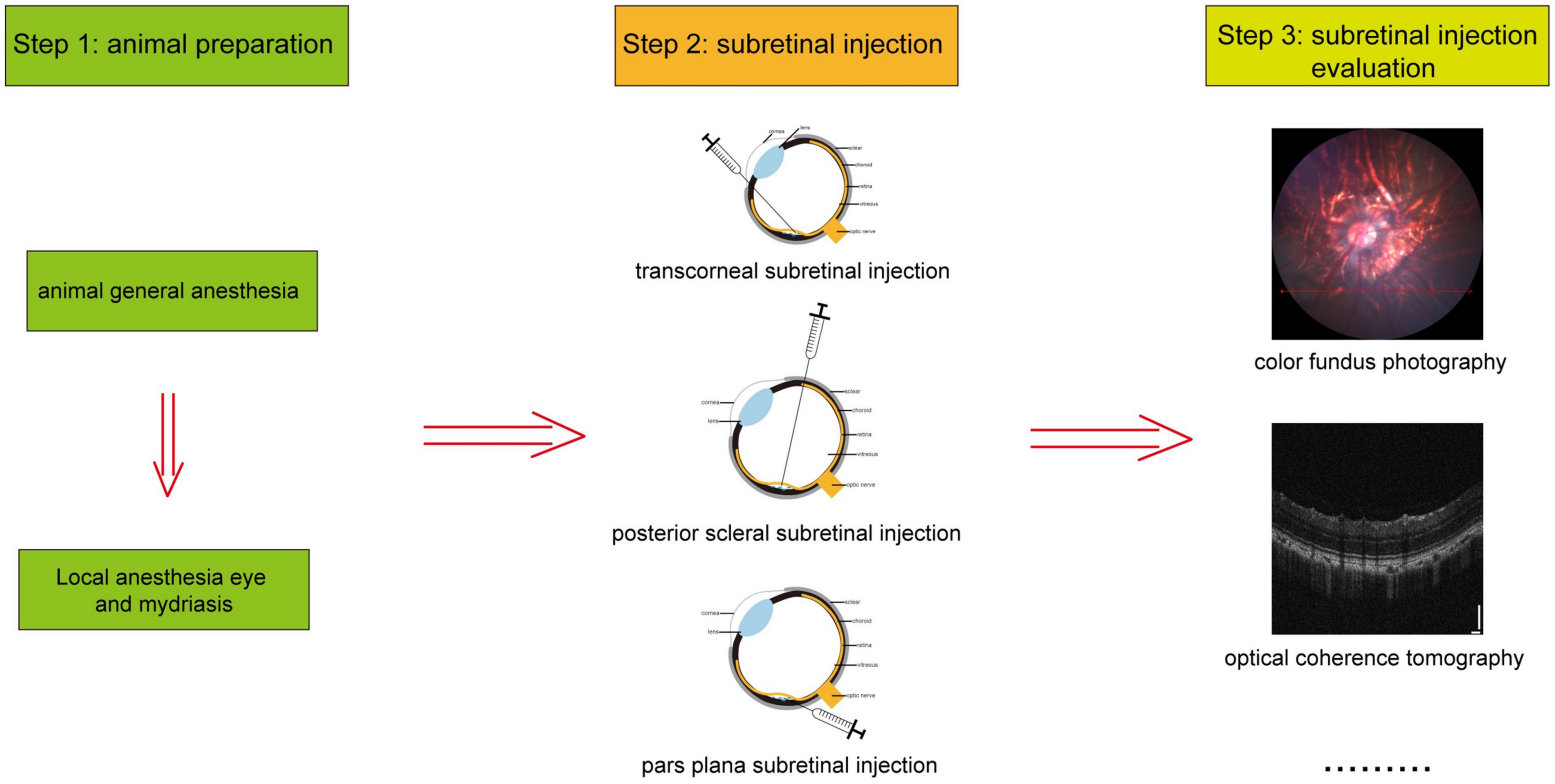
Application of subretinal injection in experimental animals.

The commonly used animal models for subretinal injection include mice, rats, guinea pigs, rabbits, and others. It has also been applied in crab-eating monkeys and Beagles, and these animal models play an important role in experimental research, which can help researchers better understand the pathogenesis of retinal diseases, assess the effectiveness of therapeutic methods, and provide theoretical support for clinical treatment. The application of subretinal injection in animal models was summarized in Figure 3.

## Application of subretinal injection in the establishment of animal disease models

The subretinal injection technique facilitates the creation of various animal disease models by administering different drugs to the subretinal space. Specifically, retinal degeneration (RD) can be modeled through the injection of sodium hyaluronate (33–36), while RPE atrophy and degeneration are induced by injecting 0.9% sodium chloride combined with bevacizumab (37, 38). An *in situ* model is also achievable by injecting a suspension of Rb-200 cells into the eyes of mice (39). Additionally, retinitis can be induced via subretinal injection of murine cytomegalovirus (MCMV) (40), and retinoblastoma (RB) can be triggered in rabbit eyes by injecting the cultured WERI-RBb-1 cell line (41). Furthermore, choroidal neovascularization (CNV) is induced in a rat model by injecting the AAV-VEGFA165 vector (42), and a uveal melanoma model is established by injecting B16 cells (43).

Boyd et al. (44) provided a summary of different approaches to subretinal injections in the most common animal model species, including rats and mice, dogs, cats, rabbits, pigs non-human primates and sheep. Subretinal injections in animal models require familiarity with the anatomy of the species used, best surgical approach, and potential complications.

## Application of subretinal injection in experimental animal therapy

In the realm of experimental animal therapy, subretinal injections have become a cornerstone in the past decade, particularly for gene therapy and the clinical treatment of retinal degenerative diseases. Such treatments have been effectively applied to conditions like age-related macular degeneration (AMD), Retinitis pigmentosa (RP) (45–47), Leber congenital amaurosis (LCA) (48), and Stargardt disease (49).

### Gene therapy

Subretinal injection plays a pivotal role in the gene therapy of various retinal diseases, as evidenced by its application in multiple animal models. For instance, in the LCA animal model, subretinal injection of recombinant AAV 2/5-OPTIRPE65 enhances the retinal resistance of RPE65 knockout mice against degeneration (50). Similarly, delivering the RPE65 gene to the retina of young and old RPE65 mutant/deficient dogs can rescue the remaining photoreceptors and aid in restoring vision (30, 51). Watanabe et al. (52) utilized 7 adeno-associated virus (AAV) serotypes in mice to illustrate the link between Crx deficiency, developmental disorders, and subsequent retinal photoreceptor degeneration. The LPCAT1-deficient mouse model exhibited electroretinogram responses and preserved retinal structure following subretinal injection of the AAV8 smCBA Lpcat1 vector (53). Moreover, subretinal injection of AAV8-GRK-Cwc27-FLAG in mutant mice improved both retinal function and morphology (54). Haldrup et al. (55) discovered that injecting a novel AAV-related RNA into a porcine choroidal neovascularization (CNV) model could inhibit CNV formation. Xu et al. (56) demonstrated that AAV2-mediated human erythropoietin, when delivered subretinally, can provide long-term protection against diabetic retinopathy in a mouse model. Furthermore, in a mouse model of Bardet-Biedl syndrome type 1 (BBS1) with severe retinal degeneration, subretinal injection of AAV-BBS1 can ameliorate the condition (57). José et al. (23) showed that subretinal injection of semiconductor polymer nanoparticles could rescue the vision of rats with retinal dystrophy. Collectively, these studies underscore the potential of subretinal injection in gene therapy, particularly for retinal degenerative and genetic defect diseases, where targeted gene delivery via vectors can restore retinal function to a significant degree.

### Cell therapy

Subretinal injection is a favored approach for stem cell transplantation in current studies, largely due to the immune-privileged status of the subretinal space, which reduces the risk of graft rejection (6, 58–68). Over recent years, this method has been extensively utilized to deliver various cell types into animal retinal models for treating retinal degenerative diseases. For instance, adult peripheral blood mononuclear cells were pre-induced and transplanted into the subretina of mice with chronic retinal degeneration (RDS) (69). Additionally, human fibroblasts engineered to express high levels of CNTF were transplanted into rats, effectively rescuing photoreceptor degeneration (70). Subretinal transplantation of RPE has been shown to inhibit laser-induced CNV (71), and the transplantation of rAAV2-CCN5 can suppress CNV and RPE fibrosis (72). Furthermore, subretinal injection of rAAV8-hGRK1-Tlcd3b has been found to overexpress Tlcd3b, rescuing retinal degeneration and significantly improving photoreceptor function in mutant mice (9). These findings have positioned progenitor and stem cells as promising candidates for subretinal treatment of retinal degeneration. These cells are believed to integrate into the retinal layers, restoring function or promoting the regeneration of various retinal cells. In particular, human embryonic stem cell-derived RPE cells delivered via subretinal injection have demonstrated safety and non-toxicity in animal models, with reports confirming feasibility and safety post-implantation, without cell migration from the scaffold or the emergence of ocular or systemic tumors (73).

## New techniques for subretinal injection

Ultra-micro subretinal injection technology, such as the 41G ultra-micro needle, is an advanced method for precise drug delivery in ophthalmic surgery, particularly for conditions like macular degeneration and in the realm of gene and stem cell therapy. This technique enables direct targeting of retinal tissue, reducing drug dispersion within the eye and prolonging the drug-retina contact time. However, due to the extremely high precision and stability required for such injections, the integration of robot-assisted technology has become a focal point in this field. A study introduced a robotic assisted subretinal injection system (RASR), which was preliminarily tested in a freshly isolated pig eye model (74). The findings indicated that robot-assisted procedures offer greater stability and reduced operator fatigue, with less tremor compared to manual injections, suggesting superior precision and operability. This enhanced visualization can boost surgical accuracy and safety while minimizing potential retinal damage. This research establishes a foundation for future advancements in ophthalmic surgical robots and the development of additional functionalities. Furthermore, another studies compared manual and robot-assisted subretinal injections through simulation experiments (75, 76). It was discovered that robotic assistance significantly enhances injection stability, decreases drift and tremor, and consequently, improves the operation’s success rate. These findings reinforce the potential of robotic technology to enhance subretinal injection surgery outcomes.

Several promising paths for the development of subretinal injection technology can be anticipated. For instance, the development of advanced robotics and AI algorithms may lead to more precise and less invasive injection techniques. Additionally, the exploration of novel biomaterials could enhance the biocompatibility and functionality of the injected cells or genes, thereby improving therapeutic outcomes. Furthermore, interdisciplinary collaborations may yield innovative solutions to address current technical challenges, such as minimizing tissue damage and enhancing cell survival post-injection. Continuous advancements in these areas will undoubtedly pave the way for more effective and efficient subretinal injection therapies in the future.

In conclusion, the fusion of ultra-micro subretinal injection technology with robotic-assisted systems is poised to significantly impact future ophthalmic surgeries, particularly those demanding high precision and stability. As this technology evolves, its application in experimental animals’ subretinal injections will be facilitated, offering standardized and reproducible drug delivery independent of injection speed (14), leading to more precise and reliable experimental outcomes.

## Conclusion

Subretinal injection is a critical technique in experimental ophthalmology, with three primary methods that are vital for animal modeling, diagnosis, and treatment. This technology has seen rapid advancements in recent years, leading to broader applications. The advent of advanced imaging and sophisticated microsurgical instruments has significantly enhanced the precision and safety of subretinal injections. Nonetheless, challenges remain, including the complexity of the procedure and the inherent risks of infection. To address these issues, operators must undergo rigorous training to ensure the consistency of each injection and to mitigate factors that could compromise the assessment of treatment efficacy or safety. Additionally, postoperative measures such as the appropriate use of antibiotics or anti-inflammatory drugs are essential to prevent infection.

It’s crucial to highlight that when translating subretinal injection techniques from experimental animals to clinical applications, particularly how to safely and effectively apply these techniques in human patients, as well as the associated risks and benefits. In translating subretinal injection techniques from experimental animals to clinical applications, current clinical challenges include precise targeting, minimizing tissue damage, and managing potential complications such as retinal detachment or infection. Solutions to these challenges involve the use of advanced imaging techniques for guidance, refined surgical instruments, and rigorous postoperative care. Researchers and clinicians must work collaboratively to overcome these hurdles and ensure the safe and effective translation of subretinal injection techniques from the laboratory to the clinic.

## References

[1] Scruggs BA Bhattarai S Helms M Cherascu I Salesevic A Stalter E. AAV2/4-RS1 gene therapy in the retinoschisin knockout mouse model of X-linked retinoschisis. PLoS One. (2022) 17:e0276298. doi: 10.1371/journal.pone.0276298, PMID: 36477475 PMC9728878

[2] Huang P Narendran S Pereira F Fukuda S Nagasaka Y Apicella I. Subretinal injection in mice to study retinal physiology and disease. Nat Protoc. (2022) 17:1468–85. doi: 10.1038/s41596-022-00689-4, PMID: 35418688 PMC11146522

[3] Petrus-Reurer S Lederer AR Baque-Vidal L Douagi I Pannagel B Khven I. Molecular profiling of stem cell-derived retinal pigment epithelial cell differentiation established for clinical translation. Stem Cell Reports. (2022) 17:1458–75. doi: 10.1016/j.stemcr.2022.05.005, PMID: 35705015 PMC9214069

[4] Ross M Obolensky A Averbukh E Ezra-Elia R Yamin E Honig H. Evaluation of photoreceptor transduction efficacy of capsid-modified adeno-associated viral vectors following Intravitreal and subretinal delivery in sheep. Hum Gene Ther. (2020) 31:719–29. doi: 10.1089/hum.2020.023, PMID: 32486858

[5] Tripepi D Jalil A Ally N Buzzi M Moussa G Rothschild PR. The role of subretinal injection in ophthalmic surgery: therapeutic agent delivery and other indications. Int J Mol Sci. (2023) 24:10535. doi: 10.3390/ijms241310535, PMID: 37445711 PMC10342145

[6] Neroeva NV Neroev VV Katargina LA Ryabina MV Ilyukhin PA Karmokova AG. Experimental stem cell replacement transplantation in retinal pigment epithelium atrophy. Vestn oftalmol. (2022) 138:7–15. doi: 10.17116/oftalma20221380317, PMID: 35801874

[7] Peng J Liang T Chen C Zhang Q Xu Y Liu J. Subretinal injection of ranibizumab in advanced pediatric vasoproliferative disorders with total retinal detachments. Graefes Arch Clin Exp Ophthalmol. (2020) 258:1005–12. doi: 10.1007/s00417-020-04600-3, PMID: 32043167

[8] Yao TT Yang Y Jin XL Wang YX Zhou YL Xu AJ. Intraocular pharmacokinetics of anti-vascular endothelial growth factor agents by intraoperative subretinal versus intravitreal injection in silicone oil-filled eyes of proliferative diabetic retinopathy: a randomized controlled pilot study. Acta Ophthalmol. (2020) 98:e795–800. doi: 10.1111/aos.14386, PMID: 32114709

[9] Qian X Liu H Fu S Lu J Hung YT Turner C. AAV8-mediated gene therapy rescues retinal degeneration phenotype in a Tlcd3b knockout mouse model. Invest Ophthalmol Vis Sci. (2022) 63:11. doi: 10.1167/iovs.63.3.11, PMID: 35275174 PMC8934561

[10] Campochiaro PA Avery R Brown DM Heier JS Ho AC Huddleston SM. Gene therapy for neovascular age-related macular degeneration by subretinal delivery of RGX-314: a phase 1/2a dose-escalation study. Lancet. (2024) 403:1563–73. doi: 10.1016/S0140-6736(24)00310-6, PMID: 38554726

[11] Gote V Sikder S Sicotte J Pal D. Ocular drug delivery: present innovations and future challenges. J Pharmacol Exp Ther. (2019) 370:602–24. doi: 10.1124/jpet.119.256933, PMID: 31072813

[12] Miyadera K Santana E Roszak K Iffrig S Visel M Iwabe S. Targeting ON-bipolar cells by AAV gene therapy stably reverses LRIT3-congenital stationary night blindness. Proc Natl Acad Sci USA. (2022) 119:e2117038119. doi: 10.1073/pnas.2117038119, PMID: 35316139 PMC9060458

[13] Peng Y Tang L Zhou Y. Subretinal injection: a review on the novel route of therapeutic delivery for vitreoretinal diseases. Ophthalmic Res. (2017) 58:217–26. doi: 10.1159/000479157, PMID: 28858866

[14] Ladha R Caspers LE Willermain F de Smet MD. Subretinal therapy: technological solutions to surgical and immunological challenges. Front Med (Lausanne). (2022) 9:846782. doi: 10.3389/fmed.2022.846782, PMID: 35402424 PMC8985755

[15] Sastry A Li JD Raynor W Viehland C Song Z Xu L. Microscope-integrated OCT-guided volumetric measurements of subretinal blebs created by a Suprachoroidal approach. Transl Vis Sci Technol. (2021) 10:24. doi: 10.1167/tvst.10.7.24, PMID: 34137836 PMC8212437

[16] Vasconcelos HM Jr Lujan BJ Pennesi ME Yang P Lauer AK. Intraoperative optical coherence tomographic findings in patients undergoing subretinal gene therapy surgery. Int J Retina Vitreous. (2020) 6:13. doi: 10.1186/s40942-020-00216-1, PMID: 32377379 PMC7193395

[17] Boyd RF Petersen-Jones SM. Techniques for subretinal injections in animals. Vet Ophthalmol. (2025) 28:506–18. 38700998 10.1111/vop.13219 PMC11911964

[18] Parikh S Le A Davenport J Gorin MB Nusinowitz S Matynia A. An alternative and validated injection method for accessing the subretinal space via a Transcleral posterior approach. J Vis Exp. (2016) 118:54808. doi: 10.3791/54808, PMID: 28060316 PMC5226358

[19] Fang Y Yao XQ Niu LL Wu JH Thee EF Chen DF. Safety evaluation of subretinal injection of trypan blue in rats. Eur Rev Med Pharmacol Sci. (2018) 22:2923–33. doi: 10.26355/eurrev_201805_15046, PMID: 29863233

[20] Guo Y Gao M Wan X Li X Wang Y Sun M. An improved method for establishment of murine retinal detachment model and its 3D vascular evaluation. Exp Eye Res. (2020) 193:107949. doi: 10.1016/j.exer.2020.107949, PMID: 32006561

[21] Comin CH Tsirukis DI Sun Y Xu X. Quantification of retinal blood leakage in fundus fluorescein angiography in a retinal angiogenesis model. Sci Rep. (2021) 11:19903. doi: 10.1038/s41598-021-99434-2, PMID: 34615975 PMC8494755

[22] Qi Y Dai X Zhang H He Y Zhang Y Han J. Trans-corneal subretinal injection in mice and its effect on the function and morphology of the retina. PLoS One. (2015) 10:e0136523. doi: 10.1371/journal.pone.0136523, PMID: 26317758 PMC4552822

[23] Maya-Vetencourt JF Manfredi G Mete M Colombo E Bramini M Di Marco S. Subretinally injected semiconducting polymer nanoparticles rescue vision in a rat model of retinal dystrophy. Nat Nanotechnol. (2020) 15:698–708. doi: 10.1038/s41565-020-0696-3, PMID: 32601447

[24] Nork TM Murphy CJ Kim CB Ver Hoeve JN Rasmussen CA Miller PE. Functional and anatomic consequences of subretinal dosing in the cynomolgus macaque. Arch Ophthalmol. (2012) 130:65–75. doi: 10.1001/archophthalmol.2011.295, PMID: 21911651 PMC3254795

[25] Bartuma H Petrus-Reurer S Aronsson M Westman S Andre H Kvanta A. In vivo imaging of subretinal bleb-induced outer retinal degeneration in the rabbit. Invest Ophthalmol Vis Sci. (2015) 56:2423–30. doi: 10.1167/iovs.14-16208, PMID: 25788649

[26] Stranak Z Ardan T Nemesh Y Toms M Toualbi L Harbottle R. Feasibility of direct vitrectomy-sparing subretinal injection for gene delivery in large animals. Curr Eye Res. (2024) 49:879–87. doi: 10.1080/02713683.2024.2343335 38666493

[27] Lai CM Estcourt MJ Himbeck RP Lee SY Yew-San Yeo I Luu C. Preclinical safety evaluation of subretinal AAV2.sFlt-1 in non-human primates. Gene Ther. (2012) 19:999–1009. doi: 10.1038/gt.2011.169, PMID: 22071974

[28] Amado D Mingozzi F Hui D Bennicelli JL Wei Z Chen Y. Safety and efficacy of subretinal readministration of a viral vector in large animals to treat congenital blindness. Sci Transl Med. (2010) 2:21ra16. doi: 10.1126/scitranslmed.3000659, PMID: 20374996 PMC4169124

[29] Barker SE Broderick CA Robbie SJ Duran Y Natkunarajah M Buch P. Subretinal delivery of adeno-associated virus serotype 2 results in minimal immune responses that allow repeat vector administration in immunocompetent mice. J Gene Med. (2009) 11:486–97. doi: 10.1002/jgm.1327, PMID: 19340848 PMC2841821

[30] Jacobson SG Acland GM Aguirre GD Aleman TS Schwartz SB Cideciyan AV. Safety of recombinant adeno-associated virus type 2-RPE65 vector delivered by ocular subretinal injection. Mol Ther. (2006) 13:1074–84. doi: 10.1016/j.ymthe.2006.03.005, PMID: 16644289

[31] Irigoyen C Amenabar Alonso A Sanchez-Molina J Rodriguez-Hidalgo M Lara-Lopez A Ruiz-Ederra J. Subretinal injection techniques for retinal disease: a review. J Clin Med. (2022) 11:4717. doi: 10.3390/jcm11164717, PMID: 36012955 PMC9409835

[32] Ross M Ofri R. The future of retinal gene therapy: evolving from subretinal to intravitreal vector delivery. Neural Regen Res. (2021) 16:1751–9. doi: 10.4103/1673-5374.306063, PMID: 33510064 PMC8328774

[33] Chidlow G Chan WO Wood JPM Casson RJ. Differential effects of experimental retinal detachment on S- and M/L-cones in rats. Mol Neurobiol. (2022) 59:117–36. doi: 10.1007/s12035-021-02582-9, PMID: 34633652

[34] Ma M Li B Zhang M Zhou L Yang F Ma F. Therapeutic effects of mesenchymal stem cell-derived exosomes on retinal detachment. Exp Eye Res. (2020) 191:107899. doi: 10.1016/j.exer.2019.107899, PMID: 31866431

[35] Choi JA Kim YJ Seo BR Koh JY Yoon YH. Potential role of zinc Dyshomeostasis in matrix Metalloproteinase-2 and -9 activation and photoreceptor cell death in experimental retinal detachment. Invest Ophthalmol Vis Sci. (2018) 59:3058–68. doi: 10.1167/iovs.17-23502, PMID: 30025117

[36] Ranty ML Carpentier S Cournot M Rico-Lattes I Malecaze F Levade T. Ceramide production associated with retinal apoptosis after retinal detachment. Graefes Arch Clin Exp Ophthalmol. (2009) 247:215–24. doi: 10.1007/s00417-008-0957-6 18958490

[37] Neroev VV Neroeva NV Zueva MV Katargina LA Tsapenko IV Ilyukhin PA. Electroretinographic signs of retinal remodeling after experimental induction of retinal pigment epithelium atrophy. Vestn oftalmol. (2021) 137:24–30. doi: 10.17116/oftalma202113704124, PMID: 34410053

[38] Narendran S Pereira F Yerramothu P Apicella I Wang SB Varshney A. A clinical metabolite of Azidothymidine inhibits experimental choroidal neovascularization and retinal pigmented epithelium degeneration. Invest Ophthalmol Vis Sci. (2020) 61:4. doi: 10.1167/iovs.61.10.4, PMID: 32749462 PMC7441363

[39] Lemaitre S Poyer F Marco S Freneaux P Doz F Aerts I. Looking for the Most suitable Orthotopic retinoblastoma mouse model in order to characterize the Tumoral development. Invest Ophthalmol Vis Sci. (2017) 58:3055–64. doi: 10.1167/iovs.17-21760 28622397

[40] Alston CI Dix RD. Reduced frequency of murine cytomegalovirus retinitis in C57BL/6 mice correlates with low levels of suppressor of cytokine signaling (SOCS)1 and SOCS3 expression within the eye during corticosteroid-induced immunosuppression. Cytokine. (2017) 97:38–41. doi: 10.1016/j.cyto.2017.05.021, PMID: 28558309 PMC5517024

[41] Nor A Diana T Tengku FA Sarina S Khairy St S Azhany Y. An in vivo study of Intravitreal Ranibizumab following subretinal inoculation of Rb cells in rabbits eyes. Cesk Slov Oftalmol. (2022) 78:112–20. doi: 10.31348/2022/13, PMID: 35760582

[42] Liu S Biesemeier AK Tschulakow AV Thakkar HV Julien-Schraermeyer S Schraermeyer U. A new rat model of treatment-naive quiescent choroidal neovascularization induced by human VEGF165 overexpression. Biol Open. (2020) 9:bio048736. doi: 10.1242/bio.048736, PMID: 32086250 PMC7295592

[43] Zhang M Lin X Zhang J Su L Ma M Ea VL. Blue light-triggered optogenetic system for treating uveal melanoma. Oncogene. (2020) 39:2118–24. doi: 10.1038/s41388-019-1119-5, PMID: 31811271

[44] Boyd RF Petersen-Jones SM. Techniques for subretinal injections in animals. Vet Ophthalmol. (2025) 28:506–18. doi: 10.1111/vop.13219, PMID: 38700998 PMC11911964

[45] Jong ED Hacibekiroglu S Guo L Sawula E Li B Li C. Soluble CX3CL1-expressing retinal pigment epithelium cells protect rod photoreceptors in a mouse model of retinitis pigmentosa. Stem Cell Res Ther. (2023) 14:212. doi: 10.1186/s13287-023-03434-0, PMID: 37605279 PMC10441732

[46] Du W Li J Tang X Yu W Zhao M. CRISPR/SaCas9-based gene editing rescues photoreceptor degeneration throughout a rhodopsin-associated autosomal dominant retinitis pigmentosa mouse model. Exp Biol Med (Maywood). (2023) 248:1818–28. doi: 10.1177/15353702231199069, PMID: 37837380 PMC10792415

[47] Ahmed CM Massengill MT Ildefonso CJ Jalligampala A Zhu P Li H. Binocular benefit following monocular subretinal AAV injection in a mouse model of autosomal dominant retinitis pigmentosa (adRP). Vis Res. (2023) 206:108189. doi: 10.1016/j.visres.2023.108189, PMID: 36773475

[48] Jo DH Jang HK Cho CS Han JH Ryu G Jung Y. Visual function restoration in a mouse model of Leber congenital amaurosis via therapeutic base editing. Mol Ther Nucleic Acids. (2023) 31:16–27. doi: 10.1016/j.omtn.2022.11.021, PMID: 36589710 PMC9792702

[49] Li R Jing Q She K Wang Q Jin X Zhao Q. Split AAV8 mediated ABCA4 expression for gene therapy of mouse Stargardt disease (STGD1). Hum Gene Ther. (2023) 34:616–28. doi: 10.1089/hum.2023.017, PMID: 37227014

[50] Georgiadis A Duran Y Ribeiro J Abelleira-Hervas L Robbie SJ Sunkel-Laing B. Development of an optimized AAV2/5 gene therapy vector for Leber congenital amaurosis owing to defects in RPE65. Gene Ther. (2016) 23:857–62. doi: 10.1038/gt.2016.66, PMID: 27653967 PMC5143366

[51] Annear MJ Mowat FM Bartoe JT Querubin J Azam SA Basche M. Successful gene therapy in older Rpe65-deficient dogs following subretinal injection of an adeno-associated vector expressing RPE65. Hum Gene Ther. (2013) 24:883–93. doi: 10.1089/hum.2013.146, PMID: 24028205

[52] Watanabe S Sanuki R Ueno S Koyasu T Hasegawa T Furukawa T. Tropisms of AAV for subretinal delivery to the neonatal mouse retina and its application for in vivo rescue of developmental photoreceptor disorders. PLoS One. (2013) 8:e54146. doi: 10.1371/journal.pone.0054146, PMID: 23335994 PMC3545928

[53] Dai X Zhang H Han J He Y Zhang Y Qi Y. Effects of subretinal gene transfer at different time points in a mouse model of retinal degeneration. PLoS One. (2016) 11:e0156542. doi: 10.1371/journal.pone.0156542, PMID: 27228218 PMC4882044

[54] Lu J Zheng KQ Bertrand RE Quinlan J Ferdous S Srinivasan T. Gene augmentation therapy to rescue degenerative photoreceptors in a Cwc27 mutant mouse model. Exp Eye Res. (2023) 234:109596. doi: 10.1016/j.exer.2023.109596 37479075

[55] Haldrup SH Fabian-Jessing BK Jakobsen TS Lindholm AB Adsersen RL Aagaard L. Subretinal AAV delivery of RNAi-therapeutics targeting VEGFA reduces choroidal neovascularization in a large animal model. Mol Ther Methods Clin Dev. (2024) 32:101242. doi: 10.1016/j.omtm.2024.101242, PMID: 38605811 PMC11007540

[56] Xu H Zhang L Gu L Lu L Gao G Li W. Subretinal delivery of AAV2-mediated human erythropoietin gene is protective and safe in experimental diabetic retinopathy. Invest Ophthalmol Vis Sci. (2014) 55:1519–30. doi: 10.1167/iovs.13-13155, PMID: 24508793

[57] Seo S Mullins RF Dumitrescu AV Bhattarai S Gratie D Wang K. Subretinal gene therapy of mice with Bardet-Biedl syndrome type 1. Invest Ophthalmol Vis Sci. (2013) 54:6118–32. doi: 10.1167/iovs.13-11673, PMID: 23900607 PMC3771708

[58] Lu B Avalos P Svendsen S Zhang C Nocito L Jones MK. GMP-grade human neural progenitors delivered subretinally protect vision in rat model of retinal degeneration and survive in minipigs. J Transl Med. (2023) 21:650. doi: 10.1186/s12967-023-04501-z, PMID: 37743503 PMC10519102

[59] Tzameret A Sher I Belkin M. Transplantation of human bone marrow mesenchymal stem cells as a thin subretinal layer ameliorates retinal degeneration in a rat model of retinal dystrophy. Exp Eye Res. (2014) 118:135–44. 24239509 10.1016/j.exer.2013.10.023

[60] Park UC Park SS Kim BH. Subretinal versus intravitreal administration of human CD34 bone marrow-derived stem cells in a rat model of inherited retinal degeneration. Ann Transl Med. (2021) 9:1275. doi: 10.21037/atm-20-4662 34532412 PMC8421968

[61] Stanzel BV Liu Z Somboonthanakij S Wongsawad W Brinken R Eter N. Human RPE stem cells grown into polarized RPE monolayers on a polyester matrix are maintained after grafting into rabbit subretinal space. Stem Cell Reports. (2014) 2:64–77. doi: 10.1016/j.stemcr.2013.11.005, PMID: 24511471 PMC3916756

[62] Tzameret A Sher I Belkin M Treves AJ Meir A Nagler A. Transplantation of human bone marrow mesenchymal stem cells as a thin subretinal layer ameliorates retinal degeneration in a rat model of retinal dystrophy. Exp Eye Res. (2014) 118:135–44. doi: 10.1016/j.exer.2013.10.023, PMID: 24239509

[63] Guan Y Cui L Qu Z Lu L Wang F Wu Y. Subretinal transplantation of rat MSCs and erythropoietin gene modified rat MSCs for protecting and rescuing degenerative retina in rats. Curr Mol Med. (2013) 13:1419–31. doi: 10.2174/15665240113139990071, PMID: 23971737

[64] Amirpour N Karamali F Rabiee F Rezaei L Esfandiari E Razavi S. Differentiation of human embryonic stem cell-derived retinal progenitors into retinal cells by sonic hedgehog and/or retinal pigmented epithelium and transplantation into the subretinal space of sodium iodate-injected rabbits. Stem Cells Dev. (2012) 21:42–53. doi: 10.1089/scd.2011.0073, PMID: 21456900

[65] Francis PJ Wang S Zhang Y Brown A Hwang T McFarland TJ. Subretinal transplantation of forebrain progenitor cells in nonhuman primates: survival and intact retinal function. Invest Ophthalmol Vis Sci. (2009) 50:3425–31. doi: 10.1167/iovs.08-2908, PMID: 19234356 PMC2826708

[66] Klassen H Kiilgaard JF Zahir T Ziaeian B Kirov I Scherfig E. Progenitor cells from the porcine neural retina express photoreceptor markers after transplantation to the subretinal space of allorecipients. Stem Cells. (2007) 25:1222–30. doi: 10.1634/stemcells.2006-0541, PMID: 17218397

[67] Wojciechowski AB Englund U Lundberg C Warfvinge K. Long-term survival and glial differentiation of the brain-derived precursor cell line RN33B after subretinal transplantation to adult normal rats. Stem Cells. (2002) 20:163–73. doi: 10.1634/stemcells.20-2-163, PMID: 11897873

[68] Kansara V Muya L Wan CR Ciulla TA. Suprachoroidal delivery of viral and nonviral gene therapy for retinal diseases. J Ocul Pharmacol Ther. (2020) 36:384–92. doi: 10.1089/jop.2019.0126, PMID: 32255727 PMC7404827

[69] Wert KJ Skeie JM Davis RJ Tsang SH Mahajan VB. Subretinal injection of gene therapy vectors and stem cells in the perinatal mouse eye. J Vis Exp. (2012) 69:4286. doi: 10.3791/4286, PMID: 23207897 PMC3578262

[70] Huang Q Xu P Xia X Hu HH Wang F Li HM. Subretinal transplantation of human fetal lung fibroblasts expressed ciliary neurotrophic factor gene prevent photoreceptor degeneration in RCS rats. Zhonghua Yan Ke Za Zhi. (2006) 42:127–30. doi: 10.3760/j:issn:0412-4081.2006.02.007 PMID: 16643727

[71] Li F Zeng Y Xu H Yin ZQ. Subretinal transplantation of retinal pigment epithelium overexpressing fibulin-5 inhibits laser-induced choroidal neovascularization in rats. Exp Eye Res. (2015) 136:78–85. doi: 10.1016/j.exer.2015.05.004, PMID: 25983185

[72] Im S Han JW Park EJ Bang JH Shin HJ Chang HS. Suppression of choroidal neovascularization and epithelial-mesenchymal transition in retinal pigmented epithelium by adeno-associated virus-mediated overexpression of CCN5 in mice. PLoS One. (2022) 17:e0269937. doi: 10.1371/journal.pone.0269937, PMID: 35696413 PMC9191714

[73] Brant Fernandes RA Koss MJ Falabella P Stefanini FR Maia M Diniz B. An innovative surgical technique for subretinal transplantation of human embryonic stem cell-derived retinal pigmented epithelium in Yucatan Mini pigs: preliminary results. Ophthalmic Surg Lasers Imaging Retina. (2016) 47:342–51. doi: 10.3928/23258160-20160324-07, PMID: 27065374

[74] Yang K Jin X Wang Z Fang Y Li Z Yang Z. Robot-assisted subretinal injection system: development and preliminary verification. BMC Ophthalmol. (2022) 22:484. doi: 10.1186/s12886-022-02720-4, PMID: 36510151 PMC9744060

[75] Ladha R Meenink T Smit J de Smet MD. Advantages of robotic assistance over a manual approach in simulated subretinal injections and its relevance for gene therapy. Gene Ther. (2023) 30:264–70. doi: 10.1038/s41434-021-00262-w, PMID: 34002047 PMC10113148

[76] L'Abbate D Prescott K Geraghty B Kearns VR Steel DHW. Biomechanical considerations for optimising subretinal injections. Surv Ophthalmol. (2024) 69:722–32. doi: 10.1016/j.survophthal.2024.05.004, PMID: 38797394

